# Hepatorenal pathologies in TNF-transgenic mouse model of rheumatoid arthritis are alleviated by anti-TNF treatment

**DOI:** 10.1186/s13075-023-03178-5

**Published:** 2023-10-02

**Authors:** Xuefei Li, Yi Wang, Ziqiang Chen, Ming Ruan, Can Yang, Maolin Zhou, Ning Li, Lianping Xing, Hao Xu, Ling Yang, Qi Shi, Yongjun Wang, Jinman Chen, Qianqian Liang

**Affiliations:** 1 Longhua Hospital, Shanghai University of Traditional Chinese Medicine, 725 Wan-Ping South Road, Shanghai, 200032 China; 2 Spine Institute, Shanghai University of Traditional Chinese Medicine, 725 Wan-Ping South Road, Shanghai, 200032 China; 3 Key Laboratory of Theory and Therapy of Muscles and Bones, Ministry of Education (Shanghai University of Traditional Chinese Medicine), 1200 Cailun Road, Shanghai, 201203 China; 4 Cardiovascular Department, Longhua Hospital, Shanghai University of Traditional Chinese Medicine, 725 Wan-Ping South Road, Shanghai, 200032 China; 5 Center for Systems Pharmacokinetics, Shanghai University of Traditional Chinese Medicine, Shanghai, 201203 China; 6 Center for Musculoskeletal Research, University of Rochester Medical Center, 601 Elmwood Ave, Box 665, Rochester, NY 14642 USA; 7 Department of Pathology & Laboratory Medicine, University of Rochester Medical Center, Rochester, NY USA

**Keywords:** Rheumatoid arthritis, Tumor necrosis factor transgenic mouse (TNF-Tg), Liver and kidney complications, Anti-TNF therapy

## Abstract

**Objective:**

To examine and quantify liver and kidney lesions and their response to anti-tumor necrosis factor (TNF) therapy in a TNF-Tg mouse model of rheumatoid arthritis (RA).

**Methods:**

Female TNF-Tg (Tg3647) mice were used as the animal model for chronic RA. Ultrasound, immunofluorescence, histological staining, serology tests, and real-time RT-PCR were used to examine the pathological changes in the liver and kidney.

**Results:**

TNF-Tg mice showed a significant decrease in the body weight and a dramatic increase in the volumes of the gallbladder, knee cavity, and popliteal lymph nodes. The liver and kidneys of TNF-Tg mice showed increased chronic inflammation and accumulation of immune cells and fibrosis, compared to wild-type (WT) mice. Moreover, upregulation of inflammatory factors and impaired normal function were observed in the liver and kidneys of TNF-Tg mice. Inflammatory infiltration and fibrosis of the liver and kidneys of female TNF-Tg mice were improved after anti-TNF treatment, and better treatment effects were achieved at 4.5-month-old mice when they were received 8 weeks of intervention.

**Conclusions:**

We found that TNF drives the development of liver and kidney pathology in female TNF-Tg mice and that there are limitations to the loss of utility of anti-TNF for the prolonged treatment of RA-associated hepatic and renal injury. This study provides a reliable and clinically relevant animal model for further studies exploring the molecular mechanisms and drug discovery for hepatorenal pathologies in RA.

**Supplementary Information:**

The online version contains supplementary material available at 10.1186/s13075-023-03178-5.

## Introduction

Autoimmune liver disease (ALD) and chronic kidney disease (CKD) are well-known extra-articular manifestations of rheumatoid arthritis (RA) and are considered RA-associated ALD (RA-ALD) and RA-associated CKD (RA-CKD), which are the leading causes of morbidity and poorer quality of life in patients with RA [1]. RA-ALD includes primary biliary cirrhosis (PBC), autoimmune hepatitis (AIH), and primary sclerosing cholangitis (PSC). PBC occurs in 1–10% of RA patients, while liver enzyme elevation and abnormal liver histology are observed in 50% and 60% of RA patients, respectively [2]. The liver damage in RA patients may progress to cirrhosis and result in a poor quality of life [1, 2].

In general, nephrotoxicity of medicine has been considered the primary cause of renal insufficiency in patients with RA. However, different opinions exist on this topic. Kanevskaia M [3] and Krel O [4] reported that CKD often presents in patients with RA prior to the use of methotrexate and targeted biologics. Approximately 37–57% of RA patients suffer from CKD, which is considered as RA-CKD. The RA-CKD pathology includes mesangial proliferative glomerulonephritis and membranous glomerulonephritis. Secondary amyloidosis and renal failure have been observed in patients with severe RA-CKD [5]. Although patients with RA are at a high risk of liver and kidney damage, leading to severe outcomes, there is a lack of animal models to study how liver and kidney injury occurs during RA, which impedes further research on the molecular mechanism underlying RA-related liver and kidney damage.

TNF-α is a critical pro-inflammatory cytokine involved in RA process [6]. As reposted, TNF-transgenic (TNF-Tg, Tg3647) mice persistently produce systemic TNF that causes inflammatory-erosive arthritis, which has been used as mouse model to study the pathology and mechanisms of RA [7–9]. TNF-Tg mice were reported to show mild ankle joint inflammation and bone erosion at 3 months of age and become more severe with aging, and anti-TNF treatment significantly alleviated both joint and lung inflammation [10–12]. TNF-α also has an important role in liver and kidney damage; it contributes to macrophages infiltration into the renal interstitium, interstitial fibrosis, and glomerulosclerosis [13, 14]. Although TNF-Tg mice have been used as animal models of chronic RA, it is still unclear whether they simulate liver and kidney damage in RA.

To better understand liver and kidney damage in patients with RA and for further drug discovery, there is an urgent need to develop a highly clinically relevant animal model. In this study, we demonstrated that female TNF-Tg mice develop hepatic perivascular and glomerular inflammation, as represented by increased numbers of immune cells around the blood vessels in the liver and glomerular areas, respectively. Inflammation infiltration in the liver and kidney of TNF-Tg mice were also confirmed by the elevated expression of inflammatory cytokines including TNF-α, IL-10, IL-1β, and IL-6. Increased fibrosis was observed in the liver parenchyma and renal interstitium of TNF-Tg mice. Moreover, inflammatory infiltration around the hepatic vessels, fibrotic areas in the liver parenchyma and renal interstitium, and glomerular diameter gallbladder volume were significantly reduced following anti-TNF therapy. Overall, we elucidated that TNF-Tg mice have different degrees of liver, gallbladder, and kidney injury of the liver and kidney, which were improved by anti-TNF therapy. This study provides an animal model to explore the pathological mechanisms of RA-related liver and kidney damage, and further drug discovery research aimed at alleviating RA-associated liver and kidney injuries.

## Materials and methods

### Animals

The present study was conducted with the approval of the Shanghai University of Traditional Chinese Medicine (SHUTCM) and the experimental animal ethics committee (ethics number PZSHUTCM201106013). TNF transgenic (TNF-Tg line 3647) mice were originally obtained from Dr. G. Kollias and back-crossed with C57BL/6 mice for more than 10 generations [7, 15]. This line of TNF-Tg mice carries one copy of the human TNF transgene and develops chronic arthritis relatively slowly. The mice were housed in an animal facility with controlled habituation and temperature, on 12-h light vs. dark cycles, and fed regular rodent chow and sterilized tap water ad libitum. Previous studies have shown that female TNF-Tg mice have an earlier onset and higher disease severity than male mice [16]. To explore the pathological changes in liver and kidney in TNF-Tg mice of different age, we divided 18 female WT mice and 18 female TNF-Tg mice into three different time points and killed the mice in 3.5, 4.5, and 5.5 months of age (*n* = 6 each time point). The body weights of the mice at 2, 3.5, 4.5, and 5.5 months were recorded.

### Anti-TNF treatment

In this experiment, 3-month-old female TNF-Tg mice were randomly divided into a model group (TNF-Tg) and an infliximab administration group (anti-TNF). Additionally, the non-transgenic litters of the same age were used as WT controls (*n* = 6 mice in each group). Mice in the anti-TNF group were administered infliximab (Cilag AG, S20171001) intraperitoneally at 10 mg/kg of body weight once a week for 8 or 12 weeks. Mice in the model and WT groups were administered an equal volume of saline in the same manner.

### B-ultrasonics

The mice were anesthetized with isoflurane (Rayward Life Technology Co., Ltd, R510-22–16), and the fur on the abdomen and legs was removed. When the mice were 3.5–5.5 months old, the abdomen, popliteal fossa, and knee joints were imaged once a month using a probe. 3D ultrasound imaging of the gallbladder, popliteal lymph nodes (PLN), and the knee joint cavity was performed using VisualSonics Vevo3100 (Fuijifilm VisualSonics, Vevo LAZR 3100). B-mode images were obtained for each animal at each time point. The volumes of the gallbladder, popliteal lymph node, and knee joint cavity were calculated using the supporting B-ultrasound analysis software.

### Tissue process

Mice were sacrificed at 3.5, 4.5, and 5.5 months old, and their livers and kidneys were harvested. Tissues were fixed in 10% phosphate-buffered formalin for 24 h, followed by serial incubation in 80%, 90%, and 100% ethanol or 10%, 20%, and 30% sucrose solutions. A series of Sects. (4 μm) were cut using Lycra Paraffin Slicer (Leica, RM2135), or 8 μm-thick sections were cut using a cryostat (Leica, CM3050S).

### Histology

For the liver and kidneys, sections from each level were stained with hematoxylin (Sigma, H-3136-25G) and eosin (Sigma, E4009-5G) (H&E), Masson (Beijing Solarbio Science & Technology Co., Ltd. G1340), and PAS (Beijing Solarbio Science & Technology Co., Ltd. G1280). The images were acquired using an Olympus VS120 microscope and × 20 objective with 0.5 NA and × 40 objective with 0.75 NA (Olympus VS120-SL). One section from each tissue sample was selected, and three random fields of per slice were quantified. The areas of inflammation and fibrosis in the liver and kidney were measured using the ImageJ software, and the average area of three fields in the same slice was used for statistical analysis.

### Immunofluorescence staining

The sections were blocked by 5% BSA and then incubated overnight at 4 °C with primary antibodies including anti-NG2 chondroitin sulfate proteoglycan (Millipore, AB5320), mouse nephrin antibody (R&D Systems, AF3159), anti-CD45R (Abcam, ab64100), anti-CD3 (Abcam, ab16669), anti-iNOS (Abcam, ab3523), and anti-F4/80 (Abcam, ab6640). After washing with phosphate buffer, the sections were incubated for 1 h at room temperature with the secondary antibodies as follows: anti-rabbit IgG (H + L), F(ab’)2 Fragment (Alexa Fluor® 488 Conjugate) (Cell signaling, #4412), anti-rat IgG (H + L) (Alexa Fluor® 488 Conjugate) (Cell signaling, #4416), anti-rabbit IgG (H + L) F(ab’)2 Fragment (Alexa Fluor® 555 Conjugate) (Cell signaling, #4413). Images were acquired using an Olympus VS120 microscope and × 20 objective with 0.5 NA and × 40 objective with 0.75 NA (Olympus VS120-SL). At least five animals were analyzed in each group, one section of each mouse was selected, and three random fields of each section were analyzed. The data were expressed as the average number of positive cells per field.

### Real-time RT-PCR analysis

Total RNA was extracted from the liver and kidneys and homogenized in TRIzol reagent (Life Technologies, Carlsbad, CA, USA). Single-stranded cDNA was generated from 1 μg of total RNA by reverse transcription using a Color Reverse Transcription Kit (with gDNA Remover) (EZB, A0010CGQ) according to the manufacturer’s instructions. Quantitative PCR was performed using 2 × SYBR Green qPCR Master Mix (EZBioscience, A0001). Data were normalized to the actin expression in each sample. The primers used are listed in Supplemental Table 1.

### Serum isolation

Peripheral blood from the mice was allowed to stand in an upright position in a 1.5-ml tube for 1 h at room temperature. The tube was then rotated at 4 °C, 3000 rpm/min for 15 min. Serum was collected from the top of the tube using a pipette and stored at − 80 °C for further experiments.

### Liver and kidney function tests

Liver and kidney functions in the mice were determined based on the concentrations of alanine aminotransferase (ALT), aspartate aminotransferase (AST), total bilirubin (TBIL), direct bilirubin (DBIL), creatinine (CREA), and urea nitrogen (UN). Two hundred microliters of peripheral blood supernatant of each mouse was extracted at different time points and sent to the Laboratory Department of Longhua Hospital, Shanghai University of Traditional Chinese Medicine, for testing.

### CES1 and DPP-IV detection

The levels of carboxylesterase-1 (CES1) were detected as previously reported [17]. An incubation reaction with a total volume of 0.1 mL was assembled with 0.002 mL of CES1 specific probe substrate NLMe, 0.005 mL serum, and 0.093 mL of PBS (pH 6.5). Serum and PBS were preincubated at 37 °C for 3 min. Luciferin was added for 10 min to initiate the luminescence reaction. Testing was performed in a white 96-well plate, and the signal value was obtained through a full-wavelength scan that represented the amount of N-alkylated d-luciferin (NL) produced by NLMe per minute, which is also the enzymatic activity of CES1. In this study, the serum CES1 levels were measured. The total CES1 activity was calculated as follows: total CES1 activity (U per liver) = specific CES1 activity (U per milligram protein) × total protein (mg/liver). The total number of activity units was calculated as follows: total activity unit (U) = metabolic rate of NLMe hydrolyzed by CES1 per minute (μmol·min^−1^).

The probe substrate GP-BAN used to detect the enzymatic activity of dipeptidyl peptidyl esterase-4 (DPP-IV) was synthesized as previously described [18]. The incubation reaction mixture, with a total volume of 0.2 mL, contained 0.002 mL GP-BAN, 0.002 mL serum, and 0.196 mL PBS (pH 7.4). Serum was preincubated with PBS at 37 °C for 3 min, and then 0.002 mL of GP-BAN was added to start the reaction. The reaction mixture was incubated at 37 °C for 20 min. To stop the reaction, 0.2 mL of ice-cold acetonitrile was added to the mixture, which was then vigorously shaken for 0.5–1 min. A black 96-well plate was used for pass detection with excitation and emission wavelengths of 430/535 nm. In this study, DPP-IV was tested in the serum. The total CES1 activity was calculated as follows: total DPP-IV activity (U per liver) = specific DPP-IV activity (U per milligram protein) × total protein (mg/liver). The total number of activity units was calculated as follows: total activity unit (U) = the metabolic rate of GP-BAN hydrolyzed by DPP-IV per minute (μmol·min^−1^).

### Statistical analysis

All statistical analyses were performed using the Statistical Product and Service Solutions (SPSS) (v21.0; IBM Corp, USA) and GraphPad Prism (version 8.0; GraphPad Software, USA). The data were expressed as the mean ± standard of deviation (SD), and differences between mean values were determined by two-tailed unpaired Student’s *t* test, one-way analysis of variance (ANOVA), or two-way ANOVA with Turkey’s multiple-comparison test using the GraphPad Prism 6 Software.

## Results

### TNF-Tg mice developed knee joint inflammation and lower body mass

TNF-Tg mice were weaker than WT mice, and the body weight of TNF-Tg mice was significantly lower than that of WT mice at 3.5, 4.5, and 5.5 months old (Supplemental Fig. 1A). As reported previously [16], the joints of female TNF-Tg mice showed pathological changes, such as knee swelling and degeneration. We used ultrasound to examined the knee joint space as well as the volume of popliteal lymph nodes (PLNs) in 3.5-, 4.5-, and 5.5-month-old mice and found that the knee cavity volumes in TNF-Tg mice increased with age and were significantly larger than those in WT mice (Supplemental Fig. 1 B-C). Increased lymph node volume in mice is a symptom of inflammatory response [19–21]. PLN volumes were significantly larger in TNF-Tg mice than those in WT mice at all time points (Supplemental Fig. 1B, D). These data suggested that the disease condition in female TNF-Tg mice simulates the disease symptoms in clinical patients with RA.

### Pathologies in the liver and kidney were significant in TNF-Tg mice

**Fig. 1:**
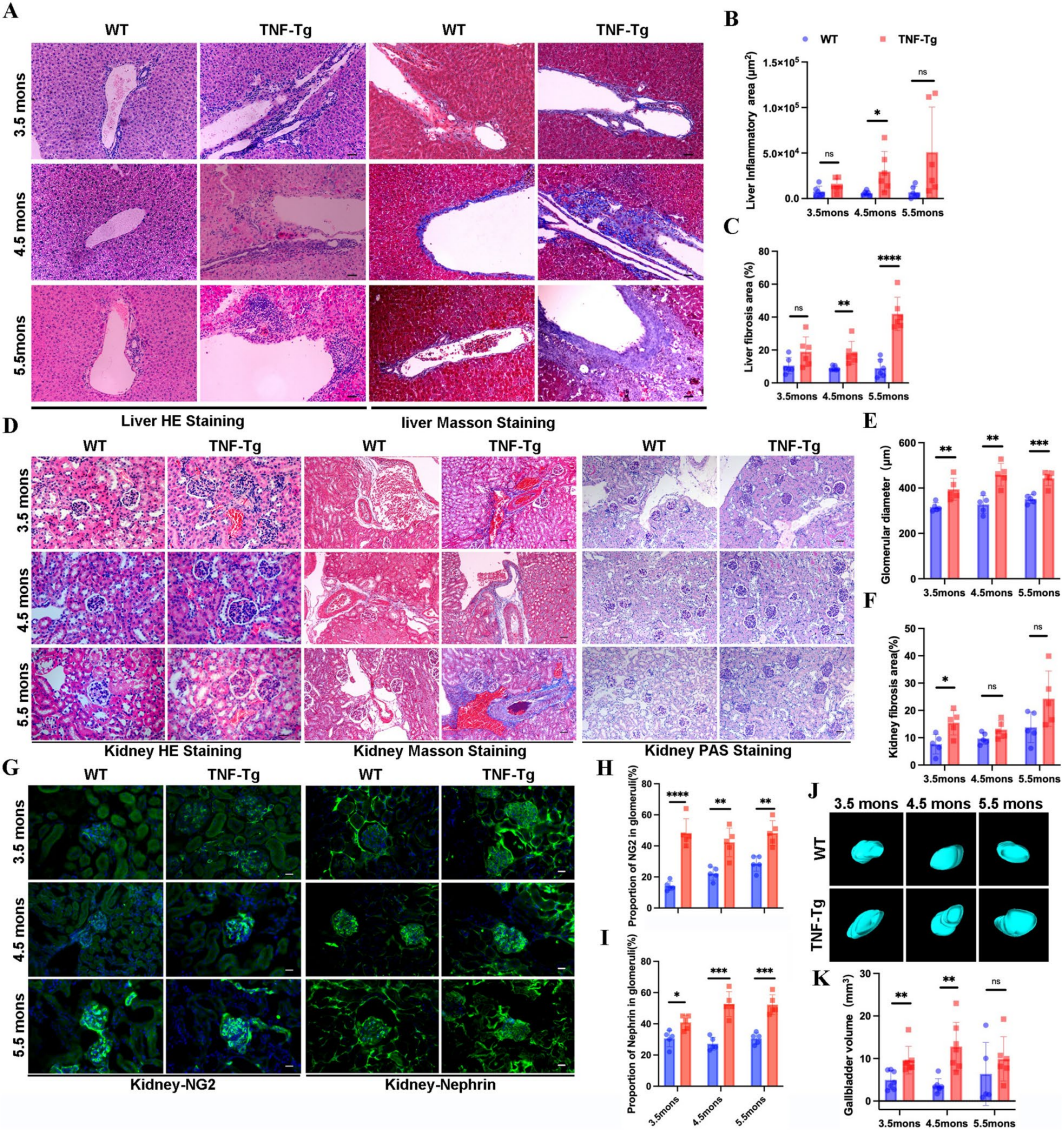
Increased fibrosis and paravascular inflammatory infiltration in the liver and aggravated interstitial and glomerular nephropathy in 3.5- to 5.5-month-old female TNF-Tg mice. **A** Representative images of the liver tissues stained with hematoxylin–eosin (left, HE staining) and Masson (right, Masson staining) (scale bar, 200 μm). **B**–**C** Quantification of the area of inflammation infiltration (**B**, statistics: *p = 0.0264) and fibrosis (**C**, statistics: **p = 0.0094; ****p < 0.0001) around the vessels. **D** Representative images of the kidney tissues with HE staining (left), Masson staining (middle), and periodic acid-Schiff staining (right). TNF-Tg kidney showed an increased number and volume of glomeruli (left), renal interstitial fibrosis (middle), and podocyte as well as mesangial proliferative (right) (scale bar, 50 μm, 200 μm). **E**–**F** Quantification of the glomerular diameter and fibrosis of the kidney showed significant proliferation of glomerulus (**E**, statistics: **p = 0.0097; **p = 0.0017; ***p = 0.0007) and renal interstitium fibrosis (**F**, statistics: *p = 0.0159) in TNF-Tg mice. **G** Representative images of the glomerulus podocyte and glomerulus mesangial of mice (scale bar, 200 μm). **H**–**I** Quantification of the percentage of NG2 (statistics: ****p < 0.0001; **p = 0.0022; **p = 0.0017) (**H**) and nephrin (statistics: *p = 0.0116; ***p = 0.0002; ***p = 0.0002) (**I**) in glomeruli. **J** Representative images of the gallbladder in mice measured by ultrasound. **K** Quantification of the volume of the gallbladder (statistics: **p = 0.0085; **p = 0.0015)

To investigate whether TNF-Tg mice have a similar pathology to clinical RA patients with concurrent liver and kidney disease, we examined the pathological changes in the livers and kidneys of these mice. We used hematoxylin and eosin (H&E) and Masson’s trichrome staining to observe inflammatory infiltration and fibrosis in the liver (Fig. 1A). A quantitative analysis showed that the inflammatory infiltration area increased significantly in the livers of 4.5-month-old TNF-Tg mice compared to that in WT mice (Fig. 1B), and the area of liver fibrosis was significantly increased in 4.5- and 5.5-month-old TNF-Tg mice (Fig. 1C). In addition, we performed HE and Masson staining of kidney sections and found that the diameters of glomeruli in TNF-Tg mice were dramatically larger than those in WT mice at each time point (Fig. 1D–E), and the fibrosis area was significantly increased in 3.5-month-old TNF-Tg mice (Fig. 1D, F). To further clarify the glomerular injury, we performed PAS staining (Fig. 1D) and labeled the podocytes and thylakoid membranes in the glomeruli by immunofluorescence (Fig. 1G). We found that the number of podocyte nuclei and thylakoid membranes in the glomeruli of TNF-Tg mice increased significantly compared to those in WT mice in all time point (Fig. 1H, I).

Among the clinical autoimmune liver diseases, primary biliary cholangitis and primary sclerosing cholangitis cause bile duct injury, which can lead to biliary stasis and result in gallbladder damage [22]. We also examined the gallbladders of TNF-Tg mice using a 3D ultrasound model and found that the gallbladder volumes of TNF-Tg mice were significantly larger than those of WT mice at 3.5 and 4.5 months of age (Fig. 1J, K). However, the wall structure of the gallbladder did not change significantly (Supplemental Fig. 1E). These results demonstrated that female TNF-Tg mice developed pathological damage in the liver, gallbladder, and kidneys, which simulates complications in clinical RA patients.

### Immunocytes and pro-inflammatory cytokines increased in the liver and kidneys of TNF-Tg mice

**Fig. 2:**
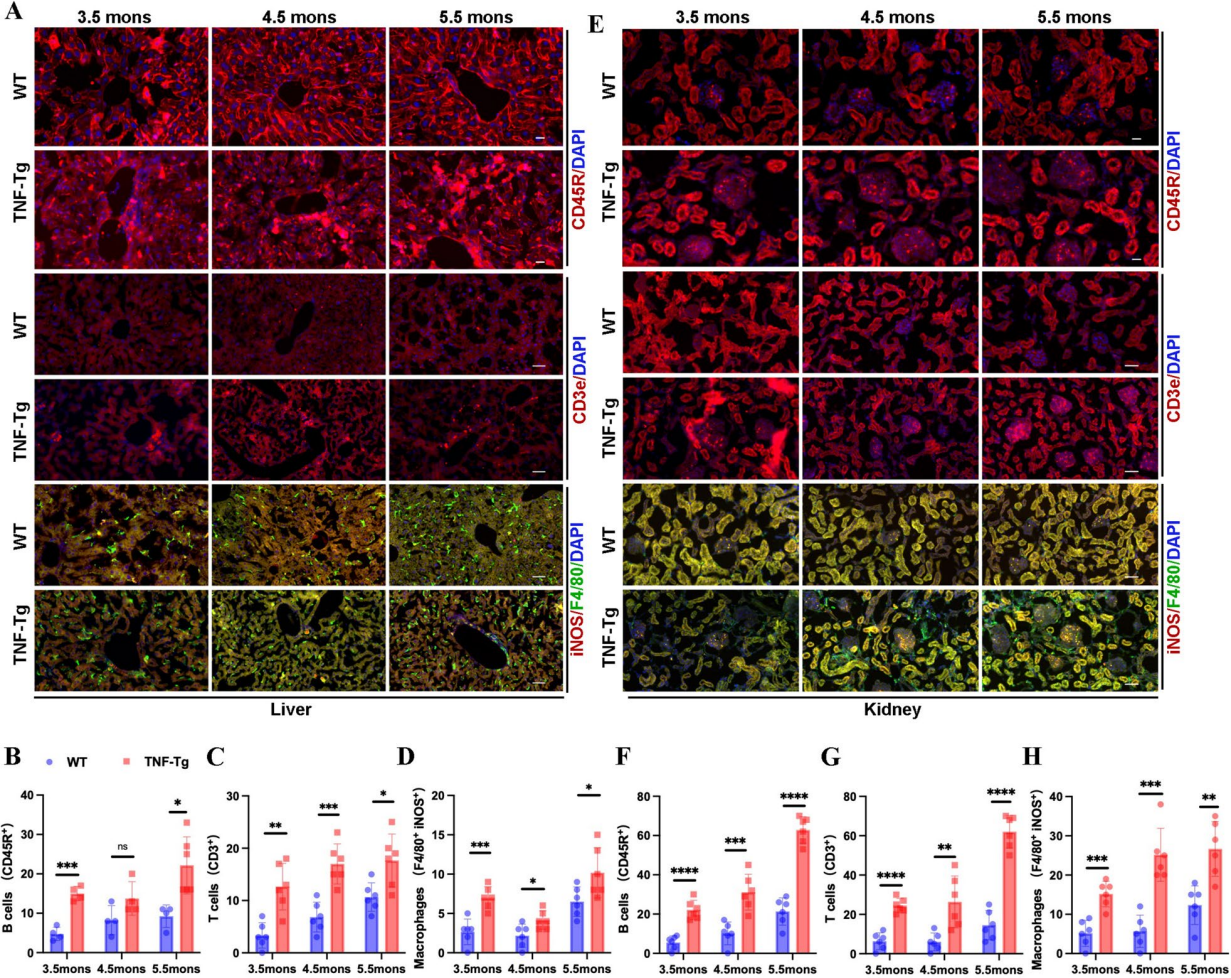
Immunocyte were significantly increased in the liver and kidney of female TNF-Tg mice. **A** Representative images of immune cells in the liver of 3.5- to 5.5- month-old female TNF-Tg mice (scale bar, 200 μm). **B**–**D** Quantification of the number of immune cells including CD45R positive B cells (**B**, statistics: ***p = 0.0002; *p = 0.0103), CD3 positive T cells (**C**, statistics: **p = 0.0011; ***p = 0.0004; *p = 0.0136), and F4/80 iNOS double positive macrophages (**D**, statistics: ***p = 0.0006; *p = 0.0262; *p = 0.0355) in the liver of TNF-Tg mice were significantly increased compared to their matched WT littermates. **E** Representative images of immune cells in the kidney of 3.5- to 5.5-month-old female TNF-Tg mice (scale bar, 200 μm). **F**–**H** Quantification of the number of immune cells including CD45R positive B cells (**F**, statistics: ****p < 0.0001; ***p = 0.0008; ****p < 0.0001), CD3 positive T cells (**G**, statistics: ****p < 0.0001; **p = 0.005; ****p < 0.0001), and F4/80 iNOS double positive macrophage (**H**, statistics: ***p = 0.0004; ***p = 0.0001; **p = 0.0021) in the kidney of TNF-Tg mice were significantly increased compared to their matched WT littermates

HE staining showed significant inflammatory infiltration; however, it is still unclear which types of immune cells accumulate in the liver and kidneys. We labeled different immune cells by immunofluorescence and found that the numbers of CD45R-positive B cells, CD3-positive T cells, and iNOS/F4-80 double-positive macrophages significantly increased and accumulated in the perivascular area of the liver (Fig. 2A–D) and inside the glomerulus (Fig. 2E–H) in TNF-Tg mice when compared to those of WT mice. These results suggested that T cells, B cells, and pro-inflammatory macrophages accumulated in the liver and kidneys of TNF-Tg mice.

**Fig. 3:**
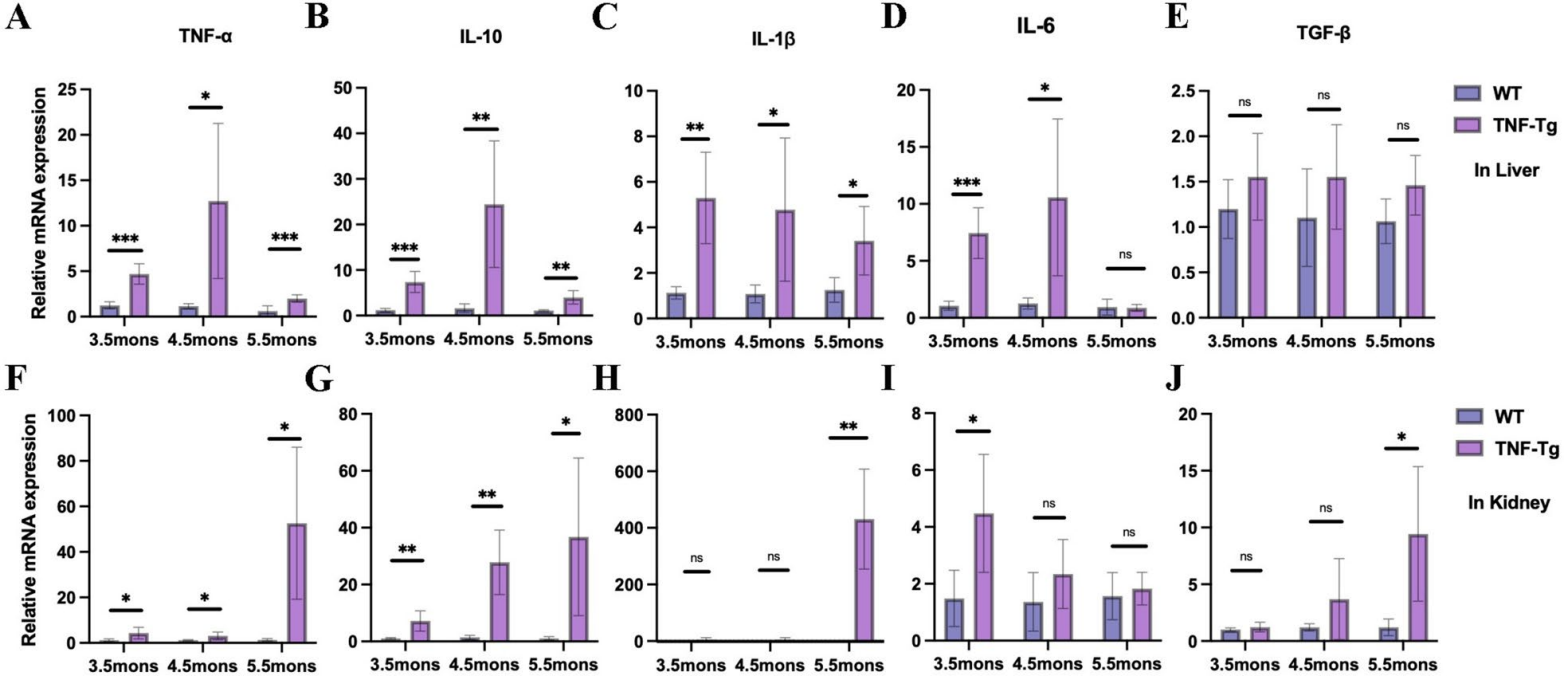
The pro-inflammatory cytokines in liver and kidney were increased in TNF-Tg mice. **A**–**E** The gene expression of inflammatory factors including TNF-α (**A**) (statistics: ***p = 0.0002; *p = 0.0163; ***p = 0.0008), IL-10 (**B**) (statistics: ***p = 0.0004; **p = 0.0054; **p = 0.0059), IL-1β (**C**) (statistics: **p = 0.0048; *p = 0.0309; *p = 0.0357), and IL-6 (**D**) (statistics: ***p = 0.0002; *p = 0.0166) were significantly elevated in the liver of TNF-Tg mice at different time points, while TGF-β (**E**) was not significantly different. **F**–**J** Intrarenal inflammatory factors TNF-α (**F**, statistics: *p = 0.0434; *p = 0.0369; *p = 0.0220) and IL-10 (**G**, statistics: **p = 0.0099; **p = 0.0011; *p = 0.0220) were significantly elevated at different time points; IL-1β (**H**, statistics: **p = 0.0092) and TGF-β (**G**, statistics: *p = 0.0297) were significantly elevated at 5.5 months of age; and IL-6 (**I**, statistics: *p = 0.0340) was significantly increased at 3.5 months of age

To further verify the infiltration of inflammation into the liver and kidneys, we examined the expression of primary pro-inflammatory cytokines. In the liver, compared to WT mice, the mRNA expression of TNF-α, IL-10, and IL-1β upregulated in TNF-Tg mice at each time point (Fig. 3A–C), while IL-6 significantly increased in 3.5- and 4.5-month-old TNF-Tg mice (Fig. 3D). However, TGF-β did not show any significantly difference (Fig. 3E).

In the kidneys, consistent with the liver, the mRNA expression of TNF-α and IL-10 increased in TNF-Tg mice at each time point (Fig. 3F, G). IL-1β and TGF-β significantly increased in 5.5-month-old mice, and IL-6 significantly increased in 3.5-month-old TNF-Tg mice (Fig. 3H–J).

### Systemic inflammation and liver damage were worsened in TNF-Tg mice

**Fig. 4:**
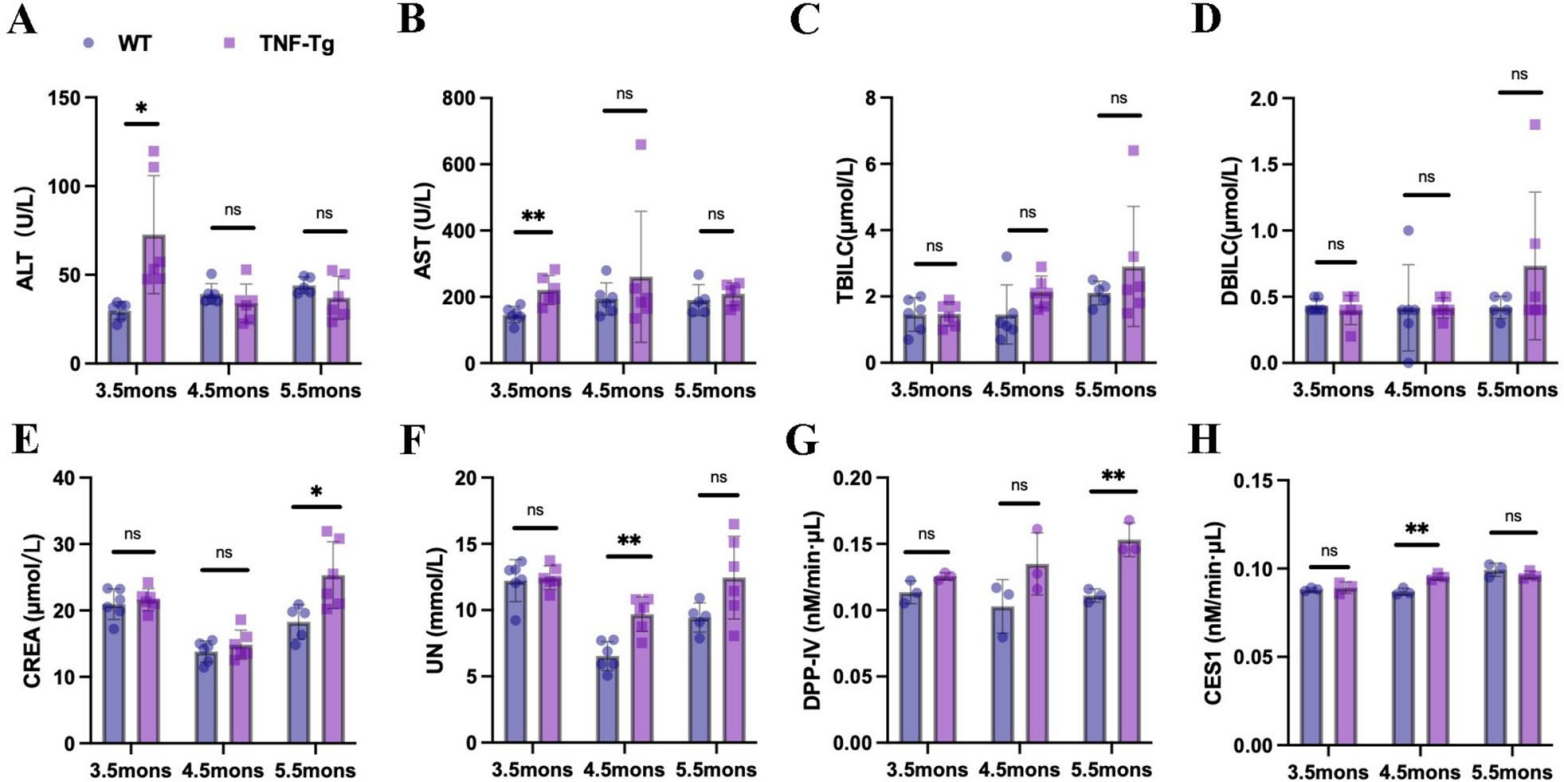
Systemic inflammation and liver damage were worsened in TNF-Tg mice. **A**–**B** Hepatic impairment was measured using blood, ALT (**A**, statistics: *p = 0.0108) and AST (**B**, statistics: **p = 0.0045) were significantly elevated in mice at 3.5 months of age. **C**–**D** There were no significant difference in TBILC (**C**) and DBILC (**D**) in the serum of the mice. **E**–**F** Creatinine (**E**, statistics: *p = 0.0201) at 5.5 months of age and urea nitrogen (**F**, statistics: **p = 0.0011) at 4.5 months of age were significantly elevated in TNF-Tg mice. **G**–**H** DPP-IV (**G**, statistics: **p = 0.0060) was significantly elevated in TNF-Tg mice at 5.5 months of age, and CES1 (**H**, statistics: **p = 0.0061) was significantly increased in mice at 4.5 months of age

Additionally, we performed liver and kidney function tests using blood samples from mice at different ages. We found that alanine aminotransferase (ALT) and aspartate aminotransferase (AST) were significantly increased in 3.5-month-old mice, creatinine (CREA) was upregulated in 5.5-month-oldmice, and urea nitrogen (UN) was increased in 4.5-month-old TNF-Tg mice (Fig. 4A–F). To further clarify the liver and kidney functions, we detected DPP-IV, an inflammatory indicator in the blood, and CES1, formed after liver injury, and found that DPP-IV increased significantly in 5.5-month-old mice (Fig. 4G), and CES1 increased significantly in 4.5-months-old-mice (Fig. 4H).

### Anti-TNF therapy reduced inflammation and fibrosis in the liver and kidneys

**Fig. 5:**
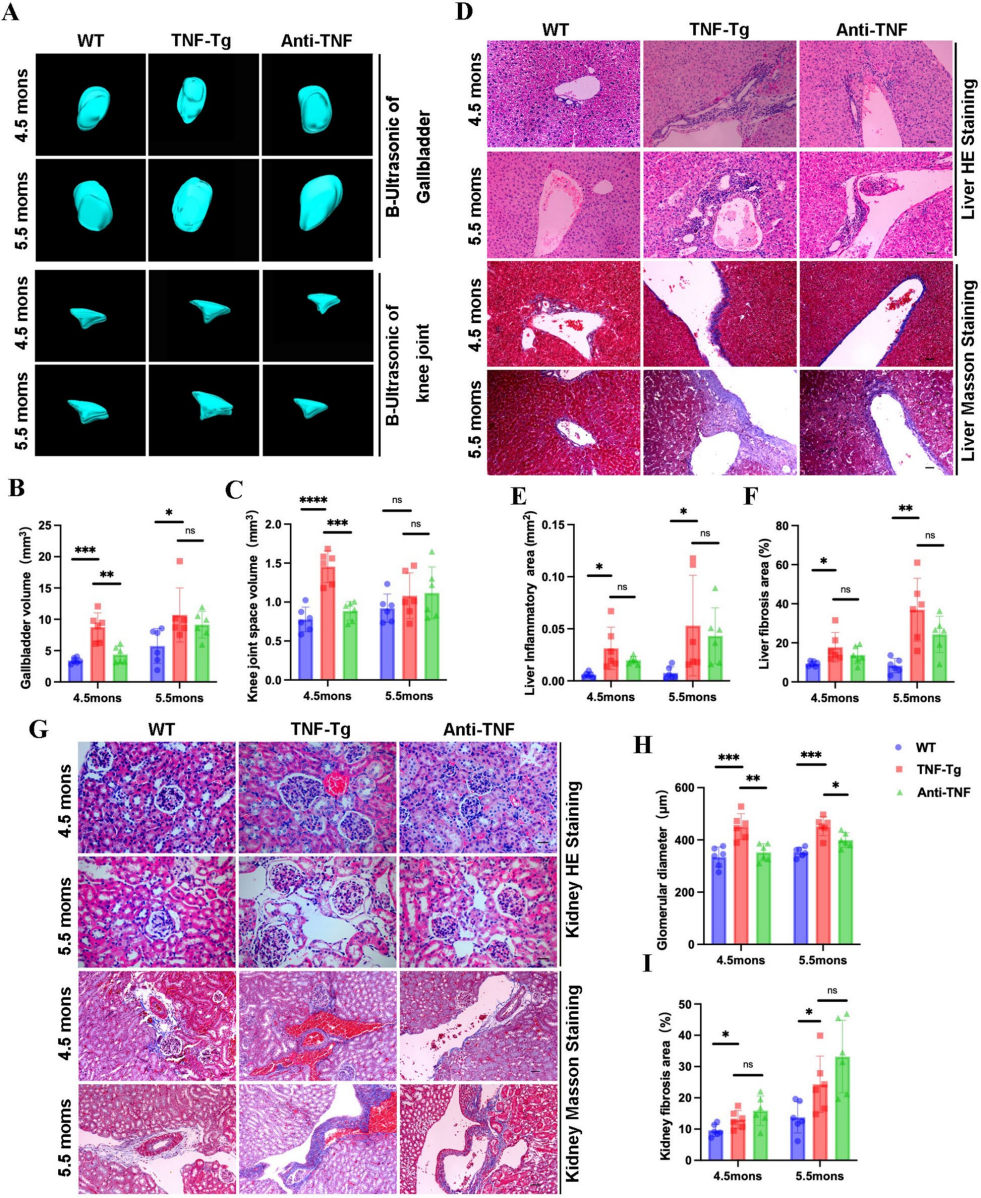
Anti-TNF therapy alleviates inflammation and fibrosis in liver and kidney. **A** Representative images of the gallbladder and the knee joint space of the mice. **B** Quantification of the volume of the gallbladder. At 4.5 months of age, anti-TNF treatment can significantly reduce gallbladder volume (anti-TNF vs. TNF-Tg, statistics: **p = 0.0022; TNF-Tg vs. WT, statistics: ***p = 0.0002; TNF-Tg vs. WT, statistics: *p = 0.0401, 5.5 months old). **C** Quantification of the volume of the knee joint space (anti-TNF vs. TNF-Tg, statistics: ***p = 0.0002; TNF-Tg vs. WT, statistics: ****p < 0.0001, 4.5 months old). **D** Representative images of the liver stained with HE and Masson. **E**–**F** Quantification of the area of hepatic inflammation (**E**) and areas of fibrosis (**F**). The hepatic inflammatory infiltration of TNF-Tg versus WT at 4.5 months old (statistics: *p = 0.0128) and at 5.5 months old (statistics: *p = 0.0447). **G** Representative images of the kidney with HE and Masson staining. **H**–**I** Quantification of glomerular diameter (**H**) showed that anti-TNF treatment decreased the glomerular diameter in TNF-Tg mice at 4.5 and 5.5 months old (statistics: **p = 0.0021; *p = 0.0177). **I** Anti-TNF did not improve the deterioration of fibrosis in the kidney. TNF-Tg versus WT at 4.5 and 5.5 months old (statistics: *p = 0.0335; *p = 0.0316)

**Fig. 6:**
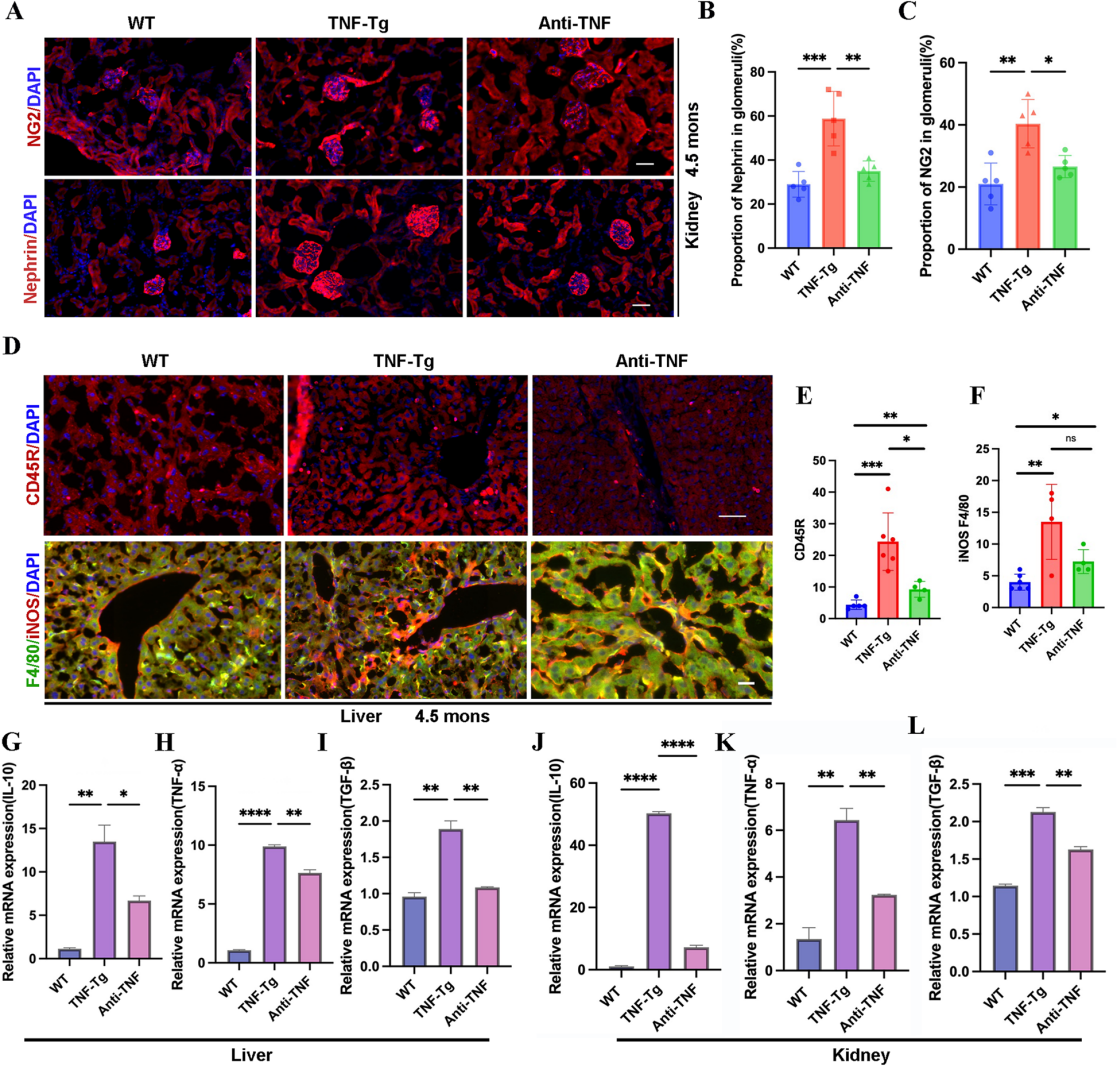
Anti-TNF therapy reduced immunocyte accumulation and pro-inflammatory cytokines in liver and kidney in TNF-Tg mice. **A** Representative images of the glomerulus podocyte and glomerulus mesangial of mice (scale bar, 50 μm). **B**–**C** Quantification of the percentage of Nephrin (Statistics: ***p = 0.0003; **p = 0.0019) (**B**) and NG2 (Statistics: **p = 0.0010; *p = 0.0119)) (**C**) in glomeruli. **D** Representative images of immune cells in the liver of 4.5-month-old female TNF-Tg mice (scale bar, 50 μm). **E**–**F** Quantification of the number of immune cells including CD45R positive B cells (**E**, statistics: ***p = 0.0010; *p = 0.0132; **p = 0.0085) and F4/80 iNOS double positive macrophage (**F**, statistics: **p = 0.0044; *p = 0.0110; ns = 0.0909) in the liver of TNF-Tg mice were significantly increased compared to their matched WT littermates, which were significantly reduced after anti-TNF treatment. **G**–**I** The gene expression of inflammatory factors in liver including IL-10 (**G**) (statistics: **p = 0.0035; *p = 0.0195), TNF-α (**H**) (statistics: ****p < 0.0001; **p = 0.0020), and TGF-β (**I**) (statistics:**p = 0.0020; **p = 0.0031) were significantly elevated in TNF-Tg mice and decreased after anti-TNF treatment. **J**-**L** The gene expression of inflammatory factors in kidney including IL-10 (**J**) (statistics: ****p < 0.0001****p < 0.0001), TNF-α (**K**) (statistics: **p = 0.0022; **p = 0.0084), and TGF-β (**L**) (statistics: ***p = 0.0004; **p = 0.0027) were significantly elevated in TNF-Tg mice and decreased after anti-TNF treatment

Anti-TNF therapy has been widely used in RA treatment; however, some patients may be insensitive to anti-TNF therapy or may gradually become unresponsive to treatment after a period of time [23, 24]. To confirm the effectiveness of anti-TNF therapy for systemic effects and the timeliness of treatment at 8 and 12 weeks of treatment, we found that the gallbladder and knee cavity volumes in TNF-Tg mice were significantly reduced after 8 weeks of treatment. However, the improvement was not significant after 12 weeks compared to those in untreated TNF-Tg mice (Fig. 5A–C). The perivascular inflammatory cell infiltration and liver fibrosis area in TNF-Tg mice were significantly higher than those in WT mice, which could not be reduced by anti-TNF treatment (Fig. 5D–F). In the kidneys, the glomerular diameter of the mice was significantly reduced after 8 and 12 weeks of treatment (Fig. 5H). However, the area of interstitial fibrosis in the kidney did not change significantly after treatment (Fig. 5I). After 8 weeks of treatment, the number of podocyte nuclei and thylakoid membranes in the glomeruli of TNF-Tg mice was reduced (Fig. 6A–C). And the effects of anti-TNF therapy on knee joint were demonstrated by decreased inflammatory infiltration and bone erosion after 8 weeks of intervention (Supplemental Fig. 1F).

### The immunocytes and pro-inflammatory cytokines in the liver and kidneys in TNF-Tg mice were mitigated by anti-TNF therapy

To better verify whether the inflammation of liver and kidney could be inhibited by anti-TNF treatment, we found a significant reduction in the number of CD45R-positive B cells and iNOS/F4-80 double-positive macrophages in the livers and kidneys after 8 weeks of intervention (Fig. 6D–F). We also examined the expression of major pro-inflammatory cytokines and found that the expression of TNF-α and IL-10 was significantly reduced in the liver and kidney after 8 weeks of treatment. Overall, anti-TNF treatment attenuated inflammation in the liver and kidneys of TNF-Tg mice after 8 weeks of treatment. However, the effects of anti-TNF therapy on fibrosis were limited (Fig. 6G–L).

## Discussion

RA is a systemic autoimmune disease, and there are many established animal models to study joint and extra-articular RA-like pathologies [25, 26]; however, the RA-related liver and kidney damage has rarely been explored. In this study, we detected the pathological changes in the liver, gallbladder, and kidney of female TNF-Tg mice, and the damage exacerbated with time. Anti-TNF treatment effectively attenuated pathological damage to the liver, gallbladder, and kidney of 4.5-month-old TNF-Tg mice.

Liver or kidney damage is a prominent extra-articular manifestation of RA; however, its etiopathogenesis remains poorly understood. Moreover, there is no preclinical model that simulates liver and kidney injury in patients with RA. In this study, we demonstrated liver and kidney inflammation in TNF-Tg mice, represented by an increased number of inflammatory and immune cells, enlargement of fibrotic regions, and upregulation of mRNA expression of pro-inflammatory cytokines. These results are consistent with the pathological changes in the liver or kidney injury in the chronic phase of RA, especially perivascular inflammatory infiltration [27]. Scientists found that overexpressed TGF-β causes a plethora of metabolic disorders and dysfunction and promotes epithelial-mesenchymal transition (EMT) and excessive deposition of ECM [5, 6], which causes fibrosis [7]. In this paper, pathological staining showed obvious fibrotic manifestations in the liver and kidney. However, there was no significant increase in the expression of TGF-β, only a tendency to increase. Therefore, the TNF-Tg mice are an ideal animal model for future studies to better understand the pathophysiological processes that may be clinically relevant to the liver and kidney complications of RA.

The clinical prevalence and the disease severity of RA demonstrated remarkable sexual dimorphism; females have higher incidence ratio versus males at least 2:1 [28]. According to a large retrospective study of more than 2 million decedent records from the National Center for Health Statistics in the USA, the data demonstrated that in decedents with RA-related inflammatory interstitial lung disease, mortality rates were increased in women compared to men [29]. As reported previously, female TNF-Tg mice have shorter lifespans, earlier onset inflammatory arthritis, and earlier onset and increased severity of inflammatory interstitial lung disease, when compared to male mice [16, 30]. In this study, we used the female TNF-Tg mice which develop more severe clinical symptom to characterize the RA-related liver and kidney pathologies, but it is interesting to design more rigorous experiments to compare the difference between gender in the future.

Liver damage in RA is confirmed in clinical practice and TNF-Tg mice. However, the early symptoms of liver damage in RA are often difficult to determine [31]. Moreover, the proteinuria levels, active urine sediments, and clinical signs cannot accurately predict renal histology in RA [32]. Routine liver and kidney function tests cannot easily detect liver and kidney injuries and inflammatory conditions in patients with RA. In our study, we found that routine peripheral blood liver and kidney function tests, including ALT, AST, TBILC, DBILC, CREA, and UN, did not respond well to liver and kidney injury or inflammation in mice. The traditional serological biomarkers, ALT and AST, provide only limited information to describe the extent of the disease and are not specific [33, 34], making it difficult to diagnose liver damage in RA. It is also possible that the degree of liver and kidney damage in our TNF-Tg mice was not severe enough, such that the difference could not be detected by routine liver and kidney function tests. Previous studies have shown that the CES1 directly reflects primary damage to hepatocytes [35] and that DPP-IV is a characteristic and mechanistic biomarker of inflammatory infiltration [36]. We tested these two indicators and found that they were elevated in 4.5- and 5.5-month-old TNF-Tg mice, which was consistent with the liver injury and inflammation observed by histology and immunofluorescence staining. Thus, DPP-IV and CES1 are promising biomarkers of liver damage in patients with RA. Future studies should devote more effort to confirming the accuracy, sensitivity, and specificity of biomarkers to aid in the diagnosis of liver and kidney injuries in patients with RA during clinical practice.

Although TNF inhibitors have been used clinically to treat RA for years [37], their effects on the liver and kidney complications of RA are still unclear. TNF plays an important role in the development of liver and kidney damage, by inducing hepatocyte apoptosis and necroptosis, liver inflammation and regeneration, and autoimmunity in the liver [38]. Moreover, high levels of TNF induce inflammatory injury in the kidney, which contributes to renal vasoconstriction by increasing superoxide generation and a subsequent reduction in the glomerular filtration rate, eventually leading to chronic kidney disease [39]. High levels of TNF are an important factor in the development of chronic inflammation as well as aging [40]. Only a few clinical studies have reported the effects of anti-TNF therapy on mitigating the PBC in patients with RA and reducing TNF-producing CD4 + T cells, serum transaminases, and immunoglobulins in patients with autoimmune hepatitis [41, 42]. We found that the accumulation of immune cells and the enlarged fibrotic areas in the liver and kidneys induced by a high level of TNF were significantly reduced by anti-TNF therapy. Thus, our findings suggest that anti-TNF therapy has the potential to improve liver and kidney pathology in RA. However, the treatment effects of anti-TNF therapy on liver and kidney damage were significant after 8 weeks of intervention when the mice were 4.5 months old, whereas it was ineffective after 12 weeks of treatment. These results are consistent with the phenomenon that some patients may gradually become unresponsive to anti-TNF treatment over time. Therefore, in patients with long-term RA, anti-TNF therapy can be combined with other approaches to achieve better clinical outcomes.

Patients with RA may have complications, such as liver and kidney damage in addition to joint lesions during the chronic phase of the disease; however, the mechanism of this is not clear. Both the disease itself and drug therapy may contribute to these complications. In our TNF-Tg mice, liver and kidney injury simulates the pathologies in RA patients, but this result from the RA disease itself or from the overexpression of TNF still needs to be further explored. Other RA mouse models, such as CIA model mice, also should be used to clarify the mechanisms of RA-complicated hepatic and renal injuries.

## Conclusion

We examined the pathological changes in the liver and kidneys of TNF-Tg mice at different time points. We found that the liver, kidneys, and gallbladder showed different degrees of inflammatory infiltration and fibrosis in TNF-Tg mice. This study demonstrates a spontaneous murine model of chronic inflammatory-erosive arthritis complicated by liver and kidney damage, which is clinically relevant in patients with RA. We provided a reliable animal model for further studies to explore the mechanisms and drug investigations of liver and kidney damage in RA.

### Supplementary Information

**Additional file 1: Supplemental Figure 1.** The knee joint volume and popliteal lymph nodes in female TNF-Tg mice were significantly increased with age, while their body weight decreased. (A) Total body weights of TNF-Tg mice were significantly lower than those of their matched WT littermates at 3.5-5.5 months of age. (**p < 0.0085; ****p < 0.0001; ***p < 0.0001). (B) Representative images of the knee joint space  and popliteal lymph nodes of the mice were acquired using ultrasound, and the results are displayed in the 3D-mode. (C) Quantification of knee joint space. Data were collected using the ultrasound software.(Statistics: ****p < 0.0001; ****p < 0.0001; *p = 0.0389). (D) Quantification of the popliteal lymph nodes volume. Data were collected using the ultrasound software. (Statistics: **p < 0.0018; ****p < 0.0001; ****p < 0.0001). (E) The representative image of HE staining of gallbladder (scale bar, 500 μm). (F) The representative image of HE staining of knee joint (scale bar, 200 μm)

**Additional file 2: Supplemental Table 1.** Primer sequences.

## Data Availability

All data are available from the corresponding authors upon reasonable request.

## Abbreviations

ALD: Autoimmune liver disease
CKD: Chronic kidney disease
RA: Rheumatoid arthritis
RA-ALD: RA-associated ALD
RA-CKD: RA-associated CKD
PBC: Primary biliary cirrhosis
AIH: Autoimmune hepatitis
PSC: Primary sclerosing cholangitis
TNF-Tg: Tumor necrosis factor-transgenic transgenic
WT: Wild-type
PLN: Popliteal lymph nodes
H&E: Hematoxylin and eosin
CES1: Carboxylesterase-1
DPP-IV: Dipeptidyl peptidyl esterase-4
ALT: Alanine aminotransferase
AST: Aspartate aminotransferase
CREA: Creatinine
UN: Urea nitrogen
TBILC: Total bilirubin
DBILC: Direct bilirubin
CIA: Collagen-induced arthritis
PCR: Polymerase chain reaction
SD: Standard deviation
ANOVA: Analysis of variance
