# Supplementary figures and images for: Hepatorenal pathologies in TNF-transgenic mouse model of rheumatoid arthritis are alleviated by anti-TNF treatment
Source: Arthritis Res Ther. 2023 Oct 2;25:188. doi: 10.1186/s13075-023-03178-5 (PMC10544221; doi:10.1186/s13075-023-03178-5)

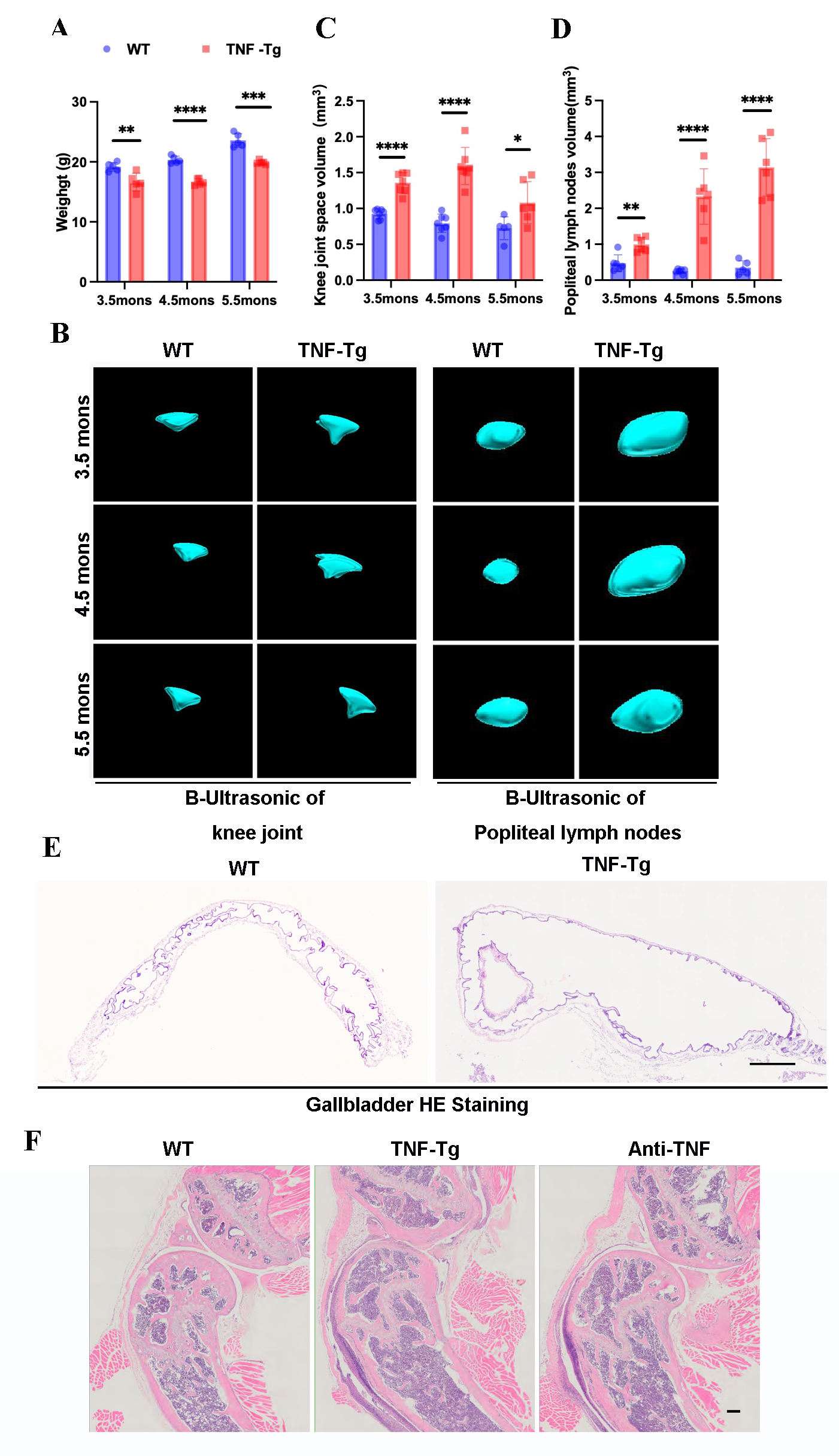

Supplement: Supplementary file 1 — Additional file 1: Supplemental Figure 1. The knee joint volume and popliteal lymph nodes in female TNF-Tg mice were significantly increased with age, while their body weight decreased. (A) Total body weights of TNF-Tg mice were significantly lower than those of their matched WT littermates at 3.5-5.5 months of age. (**p < 0.0085; ****p < 0.0001; ***p < 0.0001). (B) Representative images of the knee joint space and popliteal lymph nodes of the mice were acquired using ultrasound, and the results are displayed in the 3D-mode. (C) Quantification of knee joint space. Data were collected using the ultrasound software.(Statistics: ****p < 0.0001; ****p < 0.0001; *p = 0.0389). (D) Quantification of the popliteal lymph nodes volume. Data were collected using the ultrasound software. (Statistics: **p < 0.0018; ****p < 0.0001; ****p < 0.0001). (E) The representative image of HE staining of gallbladder (scale bar, 500 μm). (F) The representative image of HE staining of knee joint (scale bar, 200 μm) [file 13075_2023_3178_MOESM1_ESM.tif]
