# Supplementary material for: Hepatorenal pathologies in TNF-transgenic mouse model of rheumatoid arthritis are alleviated by anti-TNF treatment
Source: Arthritis Res Ther. 2023 Oct 2;25:188. doi: 10.1186/s13075-023-03178-5 (PMC10544221; doi:10.1186/s13075-023-03178-5)
Supplement: Supplementary file 2 — Additional file 2: Supplemental Table 1. Primer sequences. [file 13075_2023_3178_MOESM2_ESM.docx]

**Supplemental Table 1. Primer sequences**

| Species | Primer name | Primer sequence |
| --- | --- | --- |
| Mouse | β-Actin F | CGTTGACATCCGTAAAGACC |
| Mouse | β-Actin R | TAGGAGCCAGAGCAGTAATC |
| Mouse | TNF-α F | AGTGACAAGCCTGTAGCCC |
| Mouse | TNF-α R | GAGGTTGACTTTCTCCTGGTAT |
| Mouse | IL-10 F | GGTTGCCAAGCCTTATCGGA |
| Mouse | IL-10 R | ACCTGCTCCACTGCCTTGCT |
| Mouse | IL-1β F | CTGGTACATCAGCACCTCAC |
| Mouse | IL-1β R | AGAAACAGTCCCAGCCCATAC |
| Mouse | IL-6 F | TGTATGAACAACGATGATGCACTT |
| Mouse | IL-6 R | ACTCTGGCTTTGTCTTTCTTGTTATCT |
| Mouse | TGF-b1 F | CCGCTGCATATCGTCCTGTG |
| Mouse | TGF-b1 R | AGTGGATGGATGGTCCTATTACA |
